# Adipose-Derived Stem Cells: A Review of Signaling Networks Governing Cell Fate and Regenerative Potential in the Context of Craniofacial and Long Bone Skeletal Repair

**DOI:** 10.3390/ijms15069314

**Published:** 2014-05-26

**Authors:** Kshemendra Senarath-Yapa, Adrian McArdle, Andrea Renda, Michael T. Longaker, Natalina Quarto

**Affiliations:** 1 Hagey Laboratory for Pediatric Regenerative Medicine, Department of Surgery, School of Medicine, Stanford University, Stanford, CA 94305-2200, USA; E-Mails: kshem@stanford.edu (K.S.-Y.); amcardle@stanford.edu (A.M.); 2 Dipartimento di Scienze Biomediche Avanzate, Universita’ degli Studi di Napoli Federico II, Napoli 80131, Italy; E-Mail: renda@unina.it

**Keywords:** ASCs, osteogenesis, scaffolds, signaling, ossification, endochondral, intramembranous, bone, regeneration, stem cells

## Abstract

Improvements in medical care, nutrition and social care are resulting in a commendable change in world population demographics with an ever increasing skew towards an aging population. As the proportion of the world’s population that is considered elderly increases, so does the incidence of osteodegenerative disease and the resultant burden on healthcare. The increasing demand coupled with the limitations of contemporary approaches, have provided the impetus to develop novel tissue regeneration therapies. The use of stem cells, with their potential for self-renewal and differentiation, is one potential solution. Adipose-derived stem cells (ASCs), which are relatively easy to harvest and readily available have emerged as an ideal candidate. In this review, we explore the potential for ASCs to provide tangible therapies for craniofacial and long bone skeletal defects, outline key signaling pathways that direct these cells and describe how the developmental signaling program may provide clues on how to guide these cells *in vivo*. This review also provides an overview of the importance of establishing an osteogenic microniche using appropriately customized scaffolds and delineates some of the key challenges that still need to be overcome for adult stem cell skeletal regenerative therapy to become a clinical reality.

## 1. Introduction

Improvements in medical care and nutrition have resulted in a commendable change in the population demographics of the world and especially of developed countries. For example, over the last 50 years, the number of Americans over 65 years of age has increased from 12 to 37 million and is growing at an average rate of 2% year on year [1,2]. With an aging population however, there is a considerable increase in the incidence of osteodegenerative diseases such as osteoporosis and also the number of people suffering from fractures and a myriad of other skeletal disabilities. Furthermore, a significant need for bone reconstruction results from craniofacial skeletal pathology with over 50,000 craniectomies/craniectomies performed annually [3]. In 2001, data from the U.S. Health Cost and Utilization project reported that 12,700 cranial bone grafts were performed to repair craniofacial defects in children at a cost of over $549 million [3]. This enormous and ever increasing demand for reconstruction of skeletal defects, of long bones and the craniofacial skeleton, has provided impetus to develop novel tissue regeneration strategies with the aim of reducing reliance on existing therapies. Current approaches using autologous bone, allogeneic bone and prosthetic materials all carry significant drawbacks. Stem cells, with the promise of capacity for prolonged or even unlimited self renewal and the potential to differentiate into different cell types, have emerged as an attractivepotential solution to the challenge of bone reconstruction. Furthermore they provide the optimal strategy of replacing like with like.

In this review we explore the potential role of adult stem cells and in particular adipose-derived stem cells, a heterogeneous group of adult stem cells found in adipose tissue, that offer a suitable cell source for bone tissue regeneration in both the craniofacial skeleton and long bones. Their relative ease of harvest and abundance render this cell population as an ideal candidate for this purpose. Furthermore, this adult stem cell population is not limited by the ethical concerns that restrict the use of embryonic stem cells. Firstly, we outline how the development of the calvarium and craniofacial skeleton differs from that of the long bones. Specifically, we outline how differences in the required developmental program, in terms of signaling, may add certain nuances to the way we direct our chosen stem cell population towards bone regeneration in different anatomical locations. Attention will be given to the signaling networks that govern ASC fate and their potential to undergo osteogenic differentiation in the context of skeletal repair. We also describe how this cell population has been used in animal models of calvarial and long bone repair and the impact that this work has had or may yet have on clinical practice.

## 2. Endochondral *versus* Intramembraneous Ossification: Lessons on Signaling

The vertebrate skeleton is the product of cells from three distinct lineages. The craniofacial skeleton is formed principally from cranial neural crest cells, the axial skeleton from paraxial mesoderm and the appendicular skeleton from the lateral plate mesoderm [4]. Subsequently, in post natal life, cells with osteogenic potential called mesenchymal stem cells persist in the bone marrow and play a pivotal part in bone growth, remodeling and to some extent bone repair following injury [5]. Irrespective of the final pathway that leads to bone formation the process begins with four key processes, namely: (1) Migration; (2) Mesenchymal-epithelial interaction that results in (3) Condensation of mesenchymal cells and (4) Differentiation into either chondrogenic or osteogenic lineage depending on what is to follow [5]. Bone formation can ensue either through endochondral ossification, in which mineralized bone tissue forms via a cartilaginous anlagen, or intramembraneous ossification, in which mesenchymal cells differentiate directly into osteoblasts at ossification centers [6].

While a comprehensive account of the processes of endochondral and intramembranous ossification is beyond the scope of this review, the processes are outlined here in order to derive insights into how osteogenesis from mesenchymal cells proceeds *in situ* during development and how this may influence the way adult mesenchymal stem cells may be directed *in vivo* for the purpose of long bone and calvarial bone tissue engineering. For more detailed accounts please refer to reviews by Rice [6] and also the text from Long and Ornitz [7].

Most of the mammalian skeleton including the long bones and the axial skeleton consists of bones formed from cartilaginous templates in the process of endochondral ossification. Over the last decade studies on transgenic mice have provided insights into the molecules, that govern the key steps in this process such as mesenchymal condensation, chondrocyte differentiation, chondrocyte maturation and hypertrophy, growth plate development and osteoblastogenesis. They include the secreted proteins such as Indian Hedgehog (IHH), parathyroid hormone-related peptide (PTHrP), bone morphogenetic proteins (BMPs), wingless related integration site proteins (Wnts), fibroblast growth factors (FGFs), their receptors and several key transcriptions factors such as SRY (Sex-determining region Y) box 9 (SOX9), Runt-related transcription factor 2 (RUNX2) and Osterix (OSX).

Endochondral ossification begins with the condensation of mesenchymal cells, which is a poorly understood process during which mesenchymal cells aggregate and form clusters. Cell adhesion molecules such as neural-cell adhesion molecule (N-CAM) and neural cadherin (CDH2) have been implicated [8] as have several *Hox* genes, BMPs, and FGFs [7,9,10]. After condensation, mesenchymal cells at the center differentiate into chondrocytes that secrete cartilage matrix. Cells at the edge of the condensation form perichondrium, a layer of connective tissue which separates the developing skeletal element from the surrounding mesenchyme [7]. Several extracellular signaling pathways regulate chondrocyte differentiation and they include BMP, Wnt/β-catenin, Notch and retinoid signaling [7]. BMP signaling plays a vital role in chondrogenesis. Deletion of BMPR1A and BMPR1B, two important type 1 receptors, leads to chondrodysplasia and endochondral skeletal agenesis [11]. Wnt proteins on the other hand tend to inhibit chondrocyte differentiation in favor of osteogeneic differentiation [7]. Similarly Notch signaling and retinoid signaling plays an inhibitory role on chondrogenic differentiation [7]. At a transcriptional level, SOX9 is the key transcription factor in chondrocyte differentiation following condensation of mesenchymal cells [7]. Heterozygous mutations in the *Sox9* gene result in campomelic dysplasia, while haploinsuffuciency of SOX9 leads to chondrodysplasia in mice [12,13]. SOX9 regulates the expression of several genes that generate chondrocyte specific matrix proteins such as collagen II and aggrecan [14]. SOX9 also works in concert with two other transcription factors SOX5 and SOX6 to activate genes for cartilage specific components of the extracellular matrix in later stages of chondrocyte differentiation [7].

Chondrocyte proliferation is followed by maturation, chondrocyte hypertrophy and vascular invasion that brings in osteoprogenitors that differentiate into osteoblasts and develop primary ossification centers [15]. Vascular Endothelial Growth Factor (VEGF) produced by hypertrophic chondrocytes establish angiogenesis during longitudinal bone growth [16]. The subsequent growth and development of the long bones is a highly regulated and coordinated process in which there is coordination of chondrocyte proliferation at the epiphysis, the expanded articular end of a long bone, and their differentiation into osteoblasts at the diaphysis, the shaft of the long bone between the epiphyses. Several signaling pathways play a role in the extracellular control of chondrocyte to osteoblast differentiation and they include IHH, FGF, BMP, Wnt and Notch signaling [7]. The specific role played by these extracellular signaling pathways in governing osteoblast differentiation, in the context of endochondral ossification, is shared with osteoblast differentiation from mesenchymal progenitors through intramembraneous ossification and is therefore covered subsequently. Furthermore, there is evidence from studies in transgenic mice that certain key proteases such as matrix metalloproteinase 9 play a role in the apoptosis of hypertrophic chondrocytes, with endochondral ossification being delayed and abnormally large growth plates observed in mice lacking this enzyme [17].

The majority of the bones of the face and calvaria develop though intramembranous ossification. In this process the bones develop directly from mesenchymal cells that condense and proliferate before differentiating into osteoblasts.

Osteoblastogenesis, osteoblast differentiation during development, is controlled by a complex network at both the transcriptional level and also by extracellular signaling pathways. A key player in transcriptional control of osteoblast differentiation, Runx2, also known as Cbfa1, is essential for osteoblast differentiation [6]. Homozygous deletion of *Runx2* in mice results in complete absence of osteoblasts while haploinsufficiency of *RUNX2* in humans cause cleidocranial dysplasia [18,19]. RUNX2 is also required for appropriate functioning of osteoblasts such as bone matrix deposition and targets promoters of numerous bone proteins such as osteocalcin, bone sialoprotein, alkaline phosphatase and type 1 collagen [20,21]. Several other transcription factors, such as MAF, TAZ, SATB2, RB, GLI2, DLX5, MSX2 and BAPX1 synergize with RUNX2 or act as coactivators, while others such as TWIST1, HAND2, ZFP521, STAT1, SCHNURRI 3, GLI3, HOXA2 and the HES/HEY proteins suppress RUNX2 activity [7].

OSX is another transcription factor that is crucial for osteoblast differentiation. Deletion of *Osx* in mice in the presence of normal *Runx2* expression results in a lack of osteoblasts in mouse embryos [22]. In addition *Osx* expression is absent in *Runx2^−/−^* mice [22]. Collectively these data imply that OSX function lies downstream of RUNX2 [6]. The transcription factor OSX also plays an important role in the development and function of both osteoblast and osteocytes postnatally [23].

Several extracellular signaling pathways exert their influence on osteoblast differentiation in both endochondral and intramembranous ossification through the key transcription factors described above. These extracellular signaling pathways are outlined here.

Indian Hedgehog (IHH) signaling plays a critical role in endochondral development. Intriguingly, *Ihh^−/−^* mice lack osteoblasts within the endochondral skeletal tissue but not in intramembranous bone [24]. Without IHH, mesenchymal progenitors in the perichondrium of the cartilaginous anlagen do not express *Runx2* and therefore fail to undergo osteoblast differentiation [7]. This signaling molecule also plays a role in the generation of the trabecular bone. Mice lacking the receptor for IHH, Smoothened (Smo) lack trabecular bone [25].

Canonical Wnt signaling and more recently the non-canonical Wnt pathways have been shown to play important roles during skeletal development. Wnt ligands bind to frizzled receptors at the cell surface and LRP5 and LRP6 co-receptors. Signal transduction then proceeds with the stabilization of cytoplasmic β-catenin that enables it to translocate to the nucleus and form a transcription complex with TCF/LEF proteins [6]. LRP5 gain of function results in increased bone mass [26], whereas mutations in the gene encoding for LRP5 causes osteoporosis-pseudoglioma syndrome [27]. During the process of endochondral ossification β-catenin seems to coordinate osteoblastic differentiation from the chondrogenic anlagen. Deletion of β-catenin in OSX-positive cells causes ectopic cartilage formation rather than bone suggesting a role in this differentiation step [28]. Evidence points to a similar role during intramembranous ossification in the calvarium. Osteoblastic differentiation is blocked in calvarial mesenchymal cells, which preferentially differentiate into chondrocytes in mice with conditional deactivation of β-catenin (Dermo1-Cre) in this mesenchymal progenitor cell group [6,29]. While the role of the non-canonical pathway in these processes is less well defined it clearly plays a role. For example G-protein-coupled phosphatidylinositol signaling and PKCδ activation promotes the progression of osteoblast from RUNX2 to OSX-positive status and hence plays a role in differentiation and maturation [30].

Notch signaling suppresses osteoblast differentiation. Inhibition of the Notch pathway in the embryonic limb leads to increased bone mass and a reciprocal decrease in bone marrow mesenchymal progenitors which suggests that it plays a role in suppressing osteoblast differentiation and maintaining a larger progenitor pool [31]. Notch is thought to act by suppressing RUNX2 transcriptional activity by induction of the transcription factors HEY1 and HEYL, which physically interact with and suppress RUNX2 activity [32].

BMPs are members of the transforming growth factor-β (TGF-β) superfamily and activate the signal transduction cascade via its receptor types I and II, receptor tyrosine kinases, which upon activation phosphorylate transcription factors 1, 5 and 8 [33]. Genetic studies have demonstrated that BMP2 and BMP4 promote osteoblast differentiation by regulating the transition during the differentiation process from RUNX2 to OSX positive cells [34]. BMP3 on the other hand is thought to have a negative regulatory effect on osteogenesis with increased trabecular bone present in *Bmp3-*null mice [35]. It is thought to interact with the BMP type II receptor to inhibit BMP2 and BMP4 signaling [36].

FGF signaling has a complex and stage dependent effect on osteogenesis in endochondral and intramembranous bone development. FGF 2, 9, 18 and their corresponding receptors FGFR1-3 have all been implicated as playing important roles during skeletal development of the long bones and calvariae [37,38,39,40,41]. Ohbayashi *et al.* demonstrated that *fgf18^−/−^* embryos had defects in osteoblast maturation despite normal *Runx2* expression. In contrast FGFR1 receptor activity was shown to have a tendency to promote osteoblast differentiation at an early stage but inhibit mineralization capabilities in mature osteoblasts [42]. Furthermore gain of function mutations in FGFR1, FGFR2 and FGFR3 cause craniosynostosis while implantation of beads soaked with FGF2 and FGF4 around suture promotes osteogenic differentiation and suture closure [43,44,45]. Interestingly comparative studies looking at the differences in the osteogenic capacity of the embryonically disparate calvarial bones have shown that neural crest derived frontal bones have a superior osteogenic capacity compared to mesoderm derived parietal bone with increased activity of FGF signaling at least partly responsible for this difference [46,47]. Clearly FGF signaling plays a myriad of roles in both endochondral and intramembranous bone development but the stage at which the various components exert their effect and the integration of the different receptors and cascades need to be better deciphered.

Given the complexity of the signaling network and developmental program which is required for osteogenesis in both the endochondral and intramembranous systems it should come as no surprise that the same signaling pathways play a role in the direction of putative adult stem cells to an osteoblastic lineage for the purposes of tissue engineering.

## 3. Adipose Derived Stem Cells: The Ideal Stem Cell Candidate for Bone Tissue Engineering?

The term “stem cell” refers to a myriad of different cell types that share two key characteristics: self-renewal and the potential for differentiation into different cell types. Adult stem cells are not limited to the same extent by low availability and ethical concerns as are embryonic stem cells. They are, therefore, an appealing source for tissue engineering. Of the numerous kinds of adult stem cells that have been described in the literature two types are of particular relevance to bone tissue engineering: Bone marrow derived MSC (BMSCs) and ASCs. Bone marrow was the first source that was reported to contain MSCs [48,49]. However, isolation of these cells is associated with considerable donor site morbidity and is blighted by low yield. ASCs represent a similar cell type of multilineage potential. Rodbell and colleagues were the first to isolate these cells from rat adipose tissue in the 1960s [50]. They isolated the stromal vascular fraction after a digestion and centrifugation step [50]. In 2001 Zuk *et al.* showed using a similar protocol on human fat that the SVF of human lipoaspirates contained cells of multilineage potential that were referred to as processed lipoaspirate cells [51,52]. These cells were subsequently referred to as ASCs and have been shown to undergo adipogenesis, chondrogenesis and myogenesis *in vitro* and *in vivo* [53,54,55].

ASCs and BMSCs share many common characteristics such as multilineage differentiation capability, morphology, telomerase activity and gene expression [56]. They also share similar cell surface markers and to date a definitive profile that allows prospective isolation of ASCs has not been firmly established [2,57]. They are generally considered to be CD45^−^ CD235a^−^ CD31^−^ CD34^+^. Furthermore negativity for CD106 and positivity for CD36 distinguishes them from BM-MSCs [58]. Establishing a definitive profile is made even more difficult by the tendency for cell surface marker profile to drift during *in vitro* culture [59]. Recently Chung *et al.* used single cell-transcriptional analysis to associate low CD105 expression with increased osteogenic capacity and a combination of microfluidic analysis and FACS to identify CD90 a marker that was associated with increased osteogenic potential [60]. Selection of human ASCs on this basis delivered cells that had more osteogenic potential when studied on a calvarial defect healing model [60]. More recently, James *et al.* prospectively purified human perivascular stem cells (PSCs) from adipose tissue using FACs analysisand found a statistically significant improvement in their *in vitro* and *in vivo* osteogenic capacity relative to traditionally derived SVF [61]. This study provides an intriguing new adipose-derived cell source for skeletal regeneration [61].

## 4. Osteogenic Differentiation of ASCs and the Role of Growth Factors

As described earlier many signaling pathways converge on key transcription factors to regulate and orchestrate bone development. The same pathways such as BMP, FGF, TGF-β, Notch, Wnt/β-Catenin and Hedgehog have been shown to take part in the differentiation of mesenchymal cells, such as ASCs, into osteoblasts [2]. Delivery systems for osteoinductive growth factors, including microparticles, nanoparticle and scaffolds have therefore garnered much attention [62,63,64]. BMPs have been shown to play a significant role and numerous studies have demonstrated the potential for BMP2 to accelerate ASCs mediated bone repair when loaded onto a scaffold or when overexpressed in ASCs [55,65,66,67,68].

FGFs play vital and complex roles at all stages of both endochondral and intramembranous bone formation and, therefore, unsurprisingly have a biological function on ASCs in the context of osteogenic differentiation *in vitro* and *in vivo* [69,70].

FGF2, for example, is a well-known regulator of both osteoblastic and chondrogenic cells and acts as an autocrine/paracrine factor for bone cells. FGF2 serves as a good example of the complex ways in which FGFs can regulate bone formation [69]. Although the promotion of ASC mediated bone repair has been previously reported *in vivo*, FGF2 has also been shown to inhibit their terminal osteogenic differentiation by antagonizing the retinoic-acid mediated up-regulation of BMPR-1B [70,71,72]. This apparent paradox, however, can be explained when one considers that FGF2 promotes the proliferation and expansion of osteoprogenitor cells and, therefore, acts to maintain and expand the osteoprogenitor pool for subsequent and timely differentiation [70]. Other potential explanations lie in the fact that FGF2 can activate a plethora of different signaling pathways including MAP/ERK pathways, PKC pathways and PI3K pathways that may play different roles, such as the promotion of differentiation *versus* proliferation, depending on stage and context [69,73]. Given the apparent complexity of the role of FGF signaling, and indeed other signaling pathways, in osteoblastogenesis, the ability to regulate the activity of a particular signaling pathway, in the context of ASCs cell based therapy for skeletal regeneration, would be a huge advantage. Kwan *et al.* provided an insight into such a tunable model, demonstrating, using a system that enabled chemical control of FGF2 secretion, that this ligand would, in the correct context, promote bone regeneration *in vivo* [71].

Adequate vascularization is also a pivotal part in bone tissue regeneration. Increased angiogenesis therefore benefits bone repair [74]. It has been shown that ASCs have the capacity to differentiate *in vivo* into both osteoblasts and endothelial cells [75,76,77]. Behr *et al.* also demonstrated in a calvarial defect model that VEGFA had a more potent effect in promoting ASCs mediated calvarial regeneration than both BMP2 and FGF2 [78]. VEGFA promoted both osteogenesis and angiogenesis in this model [78].

## 5. Animal Models

The skeletal regenerative capacity of ASCs have been tested in several animal models including those for long bone defects and calvarial defects, allowing possible mimicking of the clinical condition as closely as possible.

Peterson *et al.* for example used ASCs on an osteoinductive scaffold and showed that ASCs, which were genetically modified to overexpress BMP2, were capable of healing a critical sized unicortical femoral defect [79]. The same study also showed that BMP2 alone on a scaffold was capable of significant healing thereby limiting the conclusions that can be drawn [79]. Furthermore Chou *et al.* did not find any advantage of adding ASCs onto scaffold treated with rhBMP2 in a segmental femoral defect model [80]. De Girolamo and colleagues showed that autologous ASCs-HA constructs are a potential therapy for long bone defects with full thickness defects created in the proximal epiphysis in rat tibia, while Sheyn *et al.* demonstrated that ASCs overexpressing BMP6 were capable of healing rat vertebral bone defects [81,82].

A landmark study by Cowan *et al.* revealed that a 4 mm mouse circular parietal bone defect model represents a reliable and reproducible “critically sized” defect that fails to show more than 5% healing on average over an 8 week period in the absence of any treatment modality [55]. The authors went on to show that mouse ASCs seeded on poly(lactic-*co*-glycolic acid) (PLGA) were able to achieve 80% healing [55].

Intriguingly the prospect of *in vivo* differentiation of ASCs has been raised by studies that demonstrate the ability of HA/β-TCP, apatite coated PLGA scaffold, wet-spun starch-polycaprolactone scaffold, or coral scaffold to heal critical sized calvarial defects without the need for pre differentiation. Such an advance would be of tremendous clinical importance as it would obviate the need for pretreatment of ASCs and allow reconstruction in one step without tissue leaving the operating room [67,75,83].

## 6. Scaffolds

It is well understood that stem cells reside in tightly controlled niches that can influence their behavior [2]. Scaffolds have been used to provide a suitable niche for stem cells such as ASCs when used for tissue engineering purposes, providing not only cell support but also delivering bioactive agents. Poly(d,l-lactic-coglycolic acid) (PLGA) scaffolds have been widely used, as they are biodegradable, porous, permissive for cell attachment and differentiation. The addition of a layer of hydroxyapatite (HA) can increase the bioactivity of such scaffolds and have been used successfully, as described earlier, in the treatment of critical sized calvarial defects in mice [55,84]. Polycaprolactone and hydroxyapatite nanocomposites (*n*HA)(BCP/PCL-*n*HA) have been used to drive osteogenic differentiation of ASCs through the ERK signaling pathways and others have used degradable matrices to deliver BMP4 plasmid DNA for *in vivo* genetic manipulation of cells for osteogenic differentiation [85,86]. This concept of customized scaffolds, that can be used to exert control over potential cell fate decisions of multipotent stem cells, in addition to providing a protective environment, is an important one. Levi *et al.* recently demonstrated that the provision of a suitable “osteogenic microniche”, using a hydroxyapatite-coated, BMP2 protein releasing poly-l-lactic acid (PLLA) scaffold, could direct *in vivo* differentiation of both embryonic and induced pluripotent stem cells towards an osteogenic lineage without the formation of a teratoma in the context of skeletal regeneration in a murine calvarial defect model [87].

Interestingly, it was demonstrated that the incorporation of HA onto a PLGA/PLLA scaffold stabilized β-Catenin and upregulated *Runx2* resulting in intramembranous ossification. A PLGA/PLLA scaffold alone, on the other hand, led to differentiation via endochondral ossification in mesenchymal cells derived from human embryonic stem cells. These data illustrate that appropriately devised scaffold-mediated microenvironments can manipulate the behavior of stem cells [88].

## 7. Implications of Aging for the Adult Stem Cell Pool and Regenerative Therapies

Aging has a large effect on the ability of skeletal tissue to maintain normal homeostasis and to regenerate following injury. Therefore, the implications of aging on bone physiology and also the effects of aging on resident stem cells or of any putative autologous cell population used for regenerative purposes following injury, must be considered. The progenitor pool within the bone marrow, commonly referred to as BM-MSCs, has been shown to reduce in number and to demonstrate a functional decline with age [89]. Like other somatic cells in the body, BM-MSCs are susceptible to a range of insults, including reactive oxygen species, telomere attrition and genetic mutations, that over time can accumulate and force the BM-MSCs to undergo apoptosis, enter a senescent state, or at least reduce their functional output [89,90]. Augmenting the MSCs pool with autologous ASCs is a rational therapeutic strategy to treat age-related bone disease. ASCs may improve osteogenesis directly by differentiation into osteoblasts, or indirectly through paracrine activity [68,91]. Impaired angiogenesis is associated with poor bone healing [92]. It is posited that ASCs may improve healing in tissue by increasing angiogenesis at the site of injury [93].

The stem cell pool is not immune to the effects of aging. As it will mainly be an aged population that will benefit from regenerative bone strategies, it is important to understand how aging affects the stem cells planned for autologous therapeutic use. ASCs, for example, have been shown to be susceptible to the effects of aging. They show a reduced ability to proliferate, a reduced production of angiogenic and prosurvival cytokines, and a higher susceptibility to the effects of oxidative stress [94]. Therefore, to harness the intrinsic potential of ASCs in aged individuals, we need to further understand their mechanisms and ensure that youthful cells are used in regenerative strategies. Studies have been carried out in skeletal muscle and the heart, and have identified mechanisms that can potentially rejuvenate aged cells [95,96]. This is particularly promising in the heart, where growth differentiation factor 11 was shown to reverse age-related changes. Further studies need to be carried out in ASCs to identify pathways regulating their aged program as it may have implications for their use in cell therapies for skeletal pathology [95,96].

## 8. Clinical Applications

Transitioning from preclinical research in terms of *in vivo* models to the clinical arena represents a major step. The manipulation of ASCs and human adipose-derived stromal vascular fraction (SVF) cells for therapeutic modalities must be done according to good manufacturing practices and the regulations of the FDA and/or European Medicines Agency [97]. If the cells need expansion in culture they then come under the jurisdiction of institutional Good Manufacturing Practice (GMP) guidance rules for cell manipulation. Other obstacles that must be addressed include the need for development of suitable serum free media for these cells as fetal bovine serum (FBS) is not recommended for clinical therapies given the risk of contamination and infection. Current strategies require *ex vivo* expansion of cells for two or three weeks rendering them susceptible to the possibility of genomic instability in culture [98]. Attempts at designing devices that enable a single step harvest, manipulation and grafting are therefore needed and would obviate the need for cell culture and the attendant risks of using FBS [99,100].

Despite these restrictions some examples exist for the use of ASCs based cell therapies for bone reconstruction. Lendeckel *et al.* used ASCs on a macroporous sheet together with milled autologous iliac crest bone graft to treat a large posttraumatic calvarial defect in a 7-year-old girl [101]. Thesleff and colleagues used novel scaffold consisting of β-tricalcium phosphate (β-TCP) and resorbable mesh bilaminate to achieve successful osseous reconstruction for a calvarial defect [102]. Other novel approaches include the use of a micovascular flap, used by Mesimaki *et al.* in which hASCs were used with β-TCP and BMP2 for the treatment of a large maxillary defect [103].

## 9. Future Perspectives

Despite the limited success of clinical applications for ASCs based bone reconstructions, many challenges still remain. For example, a clearer definition of the immunogenicity of ASCs and the elucidation of novel and more rigorous ways of screening population of ASCs, in terms of safety and suitability for their intended purpose, is needed [104]. Good examples of such technologies would be the enrichment for highly osteogenic subpopulations using cell sorting or FISH analysis. These processes are capable of excluding chromosomal changes that may take place in culture [104].

As the change in population demographics becomes increasingly skewed towards an aging population, the need for novel therapies to reconstruct defects of skeletal tissue becomes more pressing. ASCs represent an abundant, easily accessible and multipotent cell population that has been shown repeatedly to have the potential to undergo osteogenic differentiation *in vitro* and *in vivo* using calvarial and long bone animal models of skeletal defects. They therefore hold great promise as a source for bone tissue engineering and have rightly grabbed the attention of the scientific community. Despite the wealth of literature on this topic, the field of adult stem cells is still in its infancy. Evidence from the clinical application of these potential therapies is even more rudimentary. Therefore, much work still needs to be done before the true potential of ASCs for bone tissue reconstruction can be fully realized. Areas that deserve further attention include establishing a more comprehensive understanding of the signaling network that reliably leads to robust osteogenic differentiation and identification of a definitive cell surface marker profile. By understanding the cell surface markers that select for highly osteogenic subpopulations and developing techniques to harvest, screen and graft ASCs in one sitting, the prospect of a viable clinical approach for using ASCs in the treatment of skeletal defects will become more tangible. Furthermore the development of more sophisticated scaffolds that can recapitulate the stem cell niche and thereby modulate and guide ASCs for their desired therapeutic purpose will also be highly advantageous.
